# Biomechanical analysis of a novel bone cement bridging screw system combined with percutaneous vertebroplasty for treating Kummell’s disease

**DOI:** 10.3389/fbioe.2023.1077192

**Published:** 2023-05-18

**Authors:** Yi Zhan, Chang Bao, Huiming Yang, Liang Li, Liang Yan, Lingbo Kong, Dingjun Hao, Biao Wang

**Affiliations:** ^1^ Spine Surgery, Honghui Hospital, Xi’an Jiaotong University College of Medicine, Xi’an, Shaanxi, China; ^2^ The Second Clinical Medical College of Shaanxi University of Chinese Medicine, Xi’an, Shaanxi, China; ^3^ Department of Anaesthesiology and Perioperative Medicine, Xijing Hospital, PLA Air Force Military Medical University, Xi’an, Shaanxi, China; ^4^ Department of Orthopaedics, Shehong Municipal Hospital of TCM, Shehong, Sichuan, China

**Keywords:** Kummell’s disease, novel bone cement bridging screw, internal fixation, biomechanics analysis, sheep specimen, injury, repair, intravertebral vacuum cleft

## Abstract

Kummell’s Disease (KD) was originally proposed by Dr. Hermann Kummell in 1891 as a type of delayed posttraumatic vertebral collapse, which is a clinical phenomenon. The purpose of this experiment is to compare the strength of bone cement and the novel bone cement bridging screw in the treatment of thoracolumbar Kummell disease (KD) with other treatment methods. Thirty sheep spine specimens were selected. T12 to L2 segments were selected, and a KD intravertebral vacuum cleft model was made at the L1 segment. According to the ways of cement filling, the specimens were divided into percutaneous vertebroplasty (PVP), PVP combined with unilateral percutaneous pediculoplasty (PPP), PVP combined with bilateral PPP, unilateral novel bone cement bridging screw system combined with PVP, and bilateral cement bridging screw system combined with PVP groups. There were two experiments: three-dimensional biomechanical strength test and axial compression test. In the three-dimensional biomechanical strength test, we measured the strength of bone cement in specimens under six motion states, including flexion, extension, left bending, right bending, and left and right axial rotations. In the axial compression test, we detected the maximum axial pressure that the bone cement could withstand when it was under pressure until the bone cement was displaced. The unilateral or bilateral novel bone cement bridging screw with PVP groups had the best strength under flexion, extension, left bending, right bending, and had better biomechanical strength, with a significant difference from the other three groups (*p* < 0.05). There was no significant difference between the unilateral or bilateral novel bone cement bridging screw with PVP groups (*p* > 0.05). Unilateral and bilateral novel bone cement bridging screw could achieve similar bone cement strength. Compared with the other three groups, the unilateral or bilateral novel bone cement bridging screw with PVP groups are higher 136.35%, 152.43%; 41.93%, 51.58%; 34.37%, 43.50% respectively. The bilateral novel bone cement bridging screw with PVP could bear the largest pressure under vertical force. To conclude, the novel bone cement bridging screw can increase the strength of bone cement and avoid the loosening and displacement of bone cement in the treatment of KD of the thoracolumbar spine.

## 1 Introduction

With the advent of an aging society and the increase in the number of people with osteoporosis, Kummell’s Disease (KD) has become more common (Jiang et al., 2017; Vaccaro et al., 2017). Clinically, there is an increasing trend in the number of cases with KD. The risk of developing KD from osteoporotic vertebral fractures is approximately 13.5% (Tsujio et al., 1976). KD is characterized by the asymptomatic period of several months or even years after minor trauma to the spine of patients, recurrence of severe pain in the same region without a history of trauma, aggravation of symptoms, or even progressive kyphosis or delayed neurological deficits. KD occurs most frequently in older adults, usually affects the thoracic and lumbar spine, with T12-L1 vertebral bodies being the most commonly affected, and the male/female ratio is approximately 1:10 (Osterhouse and Kettner, 2002; Young et al., 2002).

At present, KD is difficult to treat, there is no standard treatment for KD, treatment options for KD remain controversial (Adamska et al., 2021). Surgical intervention is needed when conservative treatment is ineffective, intractable pain, a certain degree of kyphotic deformity, or neurological deficits occur (Zhang et al., 1976; Zhang et al., 2013; Feng et al., 2015; Wang et al., 2015; Lim et al., 2018). With the rapid development of minimally invasive spinal techniques, vertebroplasty and kyphoplasty have become the common surgical techniques for the treatment of KD without neurological deficits due to their advantages of minimal invasiveness, good patient tolerance, rapid pain relief, and effective kyphotic deformity correction (Peh et al., 2003; Lichti et al., 2006; Ma et al., 2010; Feng et al., 2015; Yang et al., 2015; Ateş et al., 2016; Yu et al., 2018; Lu et al., 2019).

Although satisfactory clinical results have been achieved with the use of bone cement (Feng et al., 2015; Yang et al., 2015; Meng et al., 2018), but the occurrence of the highly dangerous complications, such as loosening and displacement of bone cement during or after surgery is inevitably a cause for concern. Even if the use of bone cement could provide the patient with satisfactory short-term postoperative results, it also raises concerns about inadvertent bone cement loosening and displacement during long-term follow-up period. Once bone cement displacement occurs, complex revision surgery is often required to remove the displaced cement, reconstruct spinal stability, and restore spinal sequences. Revision surgery is difficult and carries more risks for elderly patients. In addition, elderly patients are often intolerant to revision surgery, making treatment extremely difficult.

Therefore, in order to ensure more effective interlock between bone cement and surrounding bone tissue, and avoid bone cement loosening and displacement during or after surgery, we creatively designed a novel bone cement bridging screw system to treat KD by combining with cement augmentation procedures. A new implant called stent screw-assisted internal fixation (SAIF) is also used to reconstruct vertebral stability and prevent loosening and displacement of bone cement. Unlike the novel bone cement bridging screw system we designed, SAIF will first insert a vertebral stent before inserting the screw (La Barbera et al., 2019a). After filling with bone cement, the vertebral stent can prevent the loosening and displacement of bone cement (La Barbera et al., 2019b). Currently, this technology has been used for vertebral destroyed by maliganancy and osteoporotic vertebral fractures (Cianfoni et al., 2019; Distefano et al., 2021). In our previous study, three-dimensional finite element analysis had confirmed that among the currently available treatment options for KD, the novel bone cement bridging screw system could provide the best resistance to bone cement loosening or displacement (Wang et al., 2022), but biomechanical studies are needed to further confirm its efficacy. Therefore, in the present study, we collected 30 sheep thoracolumbar vertebrae, established KD models for all specimens, and performed biomechanical analysis to investigate the strength of bone cement in different motion states and the maximum axial pressure that the bone cement can withstand after treatment with conventional modalities and the novel bone cement bridging screw system, so as to analyze whether the novel bone cement bridging screw system has the ability to prevent bone cement loosening and displacement.

## 2 Materials and methods

### 2.1 Sample collection and preparation

**FIGURE 1:**
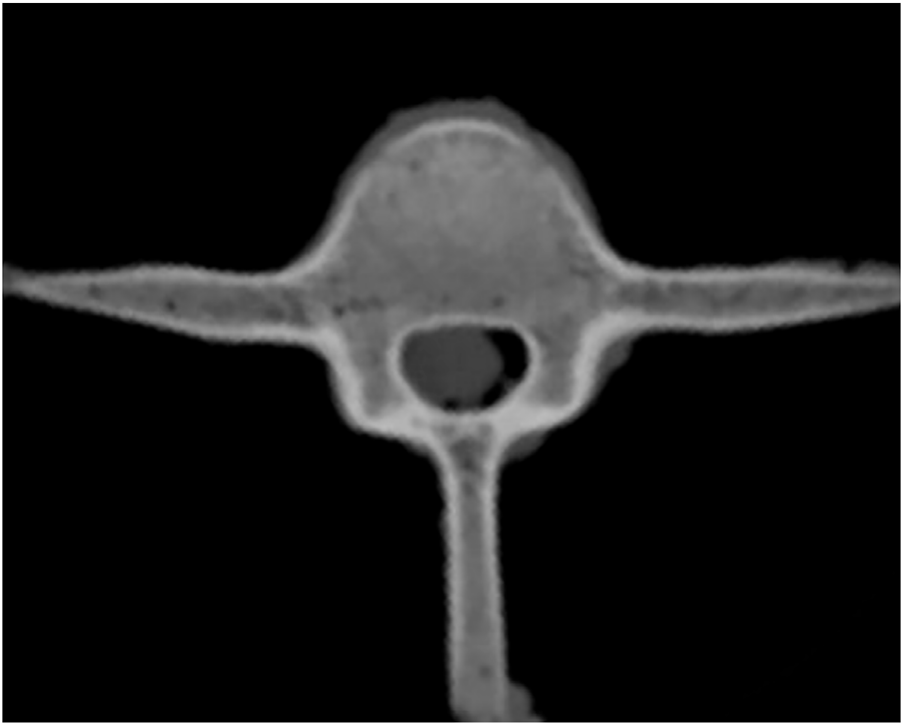
Preoperative CT scan of L1 vertebral body of sheep spine.

Thirty fresh thoracolumbar spinal segments (T12-L2) were obtained from 10-month-old sheep with an average age of 10.1 ± 0.7 months and an average weight of 45.3 ± 1.8 kg. Muscles were carefully removed from the specimens, and the posterior ligaments, joint capsules, intervertebral discs and all bony structures were preserved, then the specimens were stored in a refrigerator at −80°C for future use. Gross observation revealed no damage to all specimens. CT scans confirmed that the bony structure of the specimens was intact, without fractures, deformities, and osseous abnormalities (Figure 1).

### 2.2 Bone cement and novel bone cement bridging screw system

**FIGURE 2:**
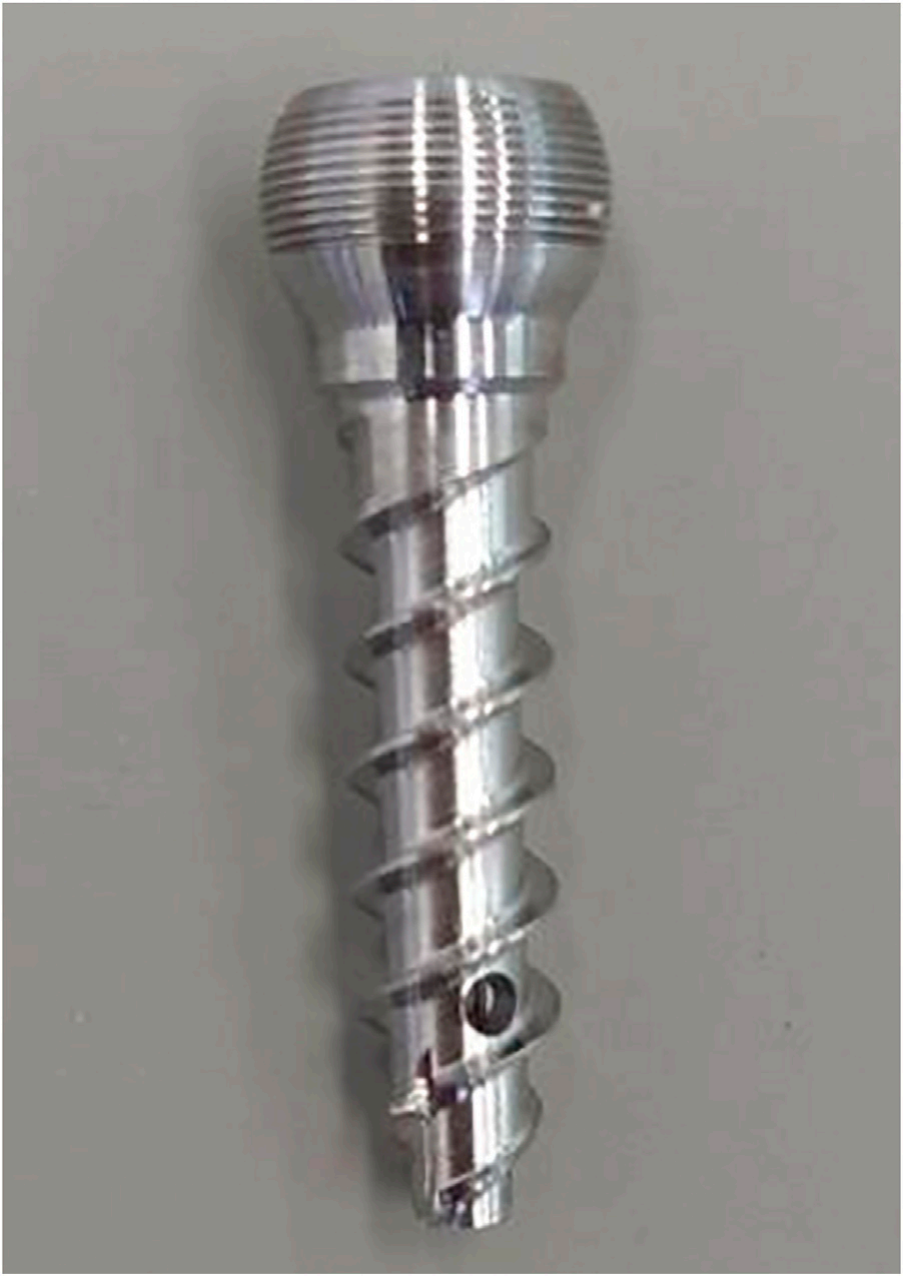
Novel bone cement bridging screw (specially made for sheep bone).

The polymethylmethacrylate (PMMA) bone cement (Tecres S.P.A., Verona, Italy), and special novel bone cement bridging screw for sheep vertebrae (diameter: 4.5 mm; length: 25 mm, Shanghai Ruizhi Medical Devices Co., Ltd., Shanghai, China) were used (Figure 2).

### 2.3 Experimental apparatus

**FIGURE 3:**
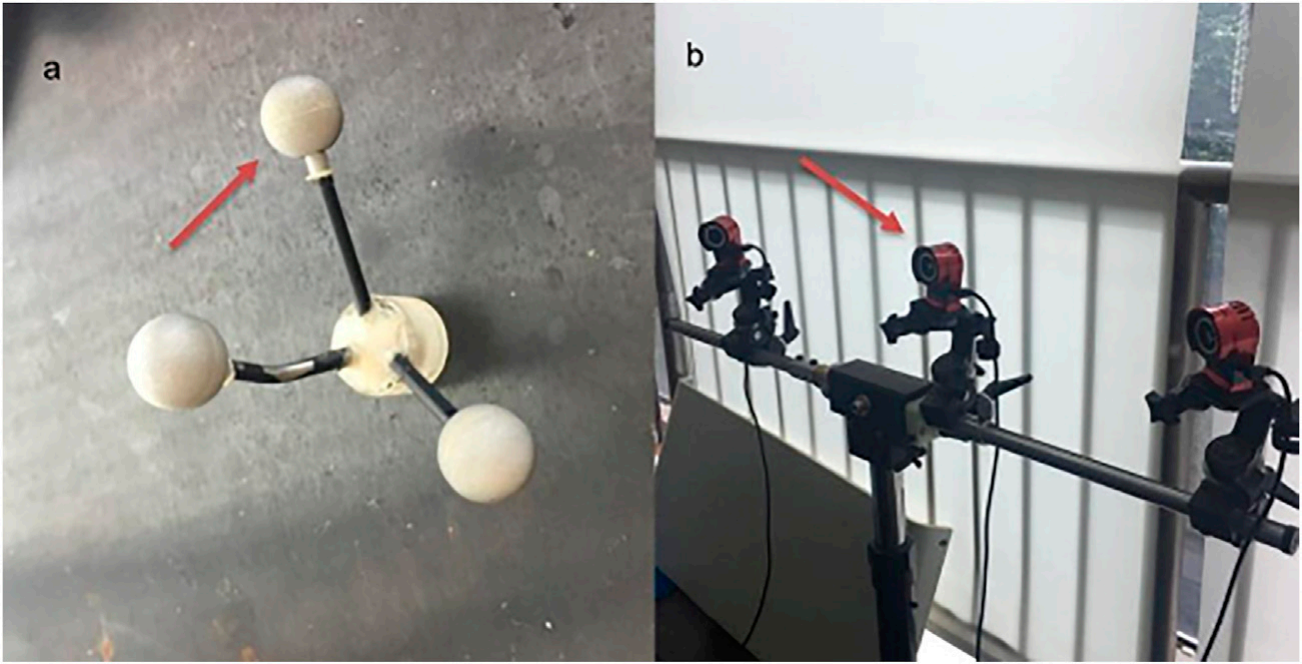
OptiTrack^®^ motion capture system: **(A)** Markers; **(B)** optiTrack^®^ motion capture system equipped behind the biomechanical machine.

1) Gemstone spectral 64-row 128-slice dual-source CT (SOMATOM Definition AS, Siemens, Healthcare GmbH, Forchheim, Germany).
2) Six-axis spinal motion testing machine (SY-03-108, Shanghai Tuoteng Biomechanics Laboratory, China). The machine is used for static testing of various metallic and non-metallic materials under flexion, extension, lateral bending and axial rotation. Parameters: maximum load 2000 N, maximum torque 50 Nm.
3) OptiTrack^®^ motion capture system (natural point, Inc. United States) equipped behind the biomechanical machine (Figure 3). The infrared camera of the system can capture the real-time movement trajectory of the light points by capturing the light point balls, which allows capture of 3-dimension motion trajectory of the specimens. The capture accuracy is 0.1 mm.
4) Microcomputer-controlled electronic universal testing machine (CMT7504, MTS, Shanghai, China). The machine is mainly used for axial tensile and compression testing of various metallic and non-metallic materials. The maximum load was 50 kN.

### 2.4 Preparation of specimens and specimen models

**FIGURE 4:**
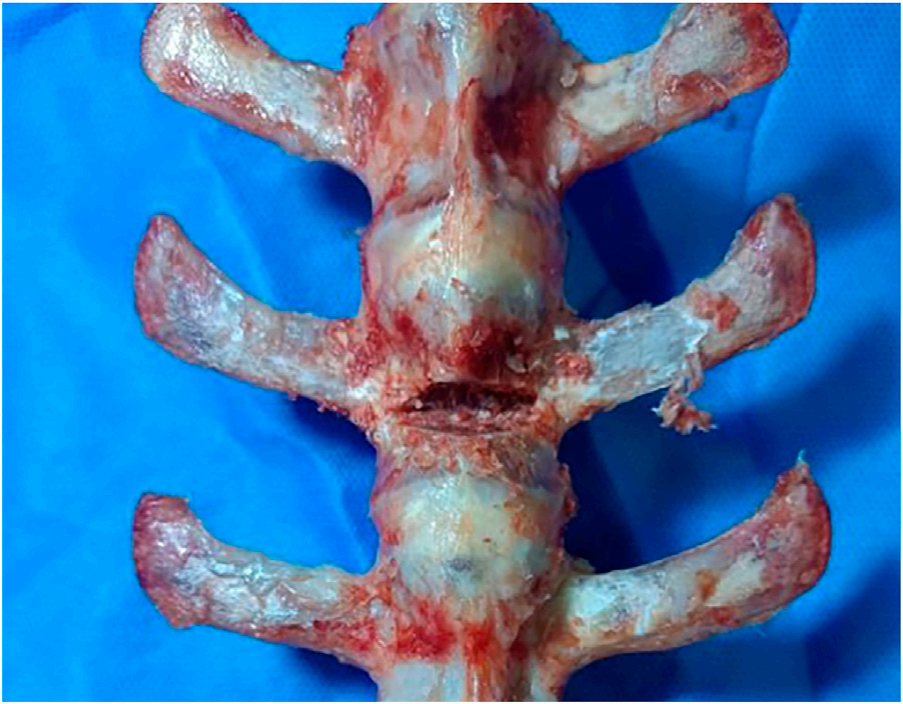
Kummell disease model.

**FIGURE 5:**
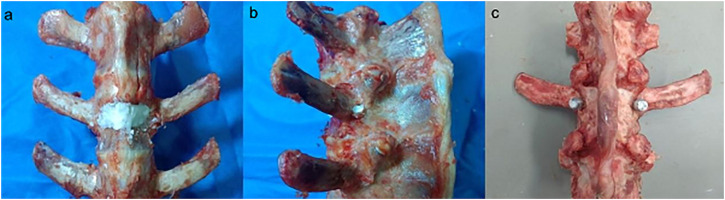
Images of specimens with different repair methods: **(A)** percutaneous vertebroplasty (PVP) alone; **(B)** PVP combined with unilateral percutaneous pediculoplasty (PPP); **(C)** bilateral novel bone cement bridging screw system combined with PVP.

The specimens were thawed at room temperature before use. The heights of anterior and posterior edges of the vertebral bodies of all specimens were measured, and the diameter of the intravertebral vacuum cleft (IVC) required to simulate KD was calculated according to the anterior and posterior vertebral body heights. Subsequently, an IVC with an angle of 15° was created by osteotomy from 1/4 below the superior endplate of L1 to 2/3 of the sagittal diameter of the vertebral body with an osteotome. Bone wax was then applied to the cancellous bone surface to simulate margin sclerosis of IVC, KD model was established (Figure 4). Based on the different ways of bone cement filling in the IVC, the specimens were randomly divided into percutaneous vertebroplasty (PVP) alone, PVP combined with unilateral percutaneous pediculoplasty (PPP), PVP combined with bilateral PPP, unilateral novel bone cement bridging screw system combined with PVP, and bilateral novel bone cement bridging screw system combined with PVP groups. Experimental models for specimens in each group were prepared (Figure 5; Table 1).

1) In the PVP alone group, with the intersection of the bottom 1/3 midline of the transverse process and the vertical line of the outer edge of superior articular process as the puncture point, a 2.0 mm-diameter drill was used to create a bony channel by drilling along the central axis of the pedicle at a medial inclination angle of 20°–25° to the spinous process. The bony channel reached 80% of the anterior edge of the vertebral body, and entered the IVC in the anterior edge of the vertebral body. Then bone cement was prepared by mixing PMMA powder and liquid in a 2:1 ratio, which was then injected into the channel until bone cement had reached a toothpaste-like viscosity. Bone cement injection was stopped after the IVC was completely filled with bone cement, and the injection device was removed after the cement solidified.
2) In the PVP combined with unilateral PPP group, imitate the method in 1) to make a KD model. PVP was performed as described for the PVP alone group. After vertebroplasty was completed, injection device was slowly retracted while the bone cement was slowly injected until pediculoplasty was completed. Finally, the injection device was removed after the cement solidified.
3) In the PVP combined with bilateral PPP group, performed as described for the PVP combined with unilateral PPP group, the difference is that this group performs PPP on both sides.
4) In the unilateral novel bone cement bridging screw system combined with PVP group, with the intersection of the bottom 1/3 midline of the transverse process and the vertical line of the outer edge of superior articular process as the puncture point, a 2.0 mm-diameter drill was used to create a bony screw channel by drilling along the central axis of the pedicle at a medial inclination angle of 20°–25° to the spinous process. The bony screw channel reached 80% of the anterior edge of the vertebral body, and entered the IVC. A ball-tip probe was used to explore whether the four walls of the screw channel was intact. After tapping the screw channel with a 2.0 mm-diameter tap, screw channel was probed with a probe again. The novel bone cement screw was then screwed into the channel to ensure that the bone cement outlets on the front of the screw were located in the wedge-shaped cavity. Then bone cement was prepared by mixing PMMA powder and liquid in a 2:1 ratio. After bone cement had reached a toothpaste-like viscosity, bone cement was injected in the novel bone cement screw until the vertebroplasty was completed and the IVC was completely filled with bone cement.
5) In the bilateral novel bone cement bridging screw system combined with PVP group, performed as described for the unilateral novel bone cement bridging screw system combined with PVP group, the difference is that this group performs novel bone cement bridging screw on both sides.

**TABLE 1:** Grouping of sheep spine specimens.

Group	The ways of bone cement filling	Experiment
A1	PVP alone	Three-dimensional biomechanical strength test
B1	PVP combined with unilateral PPP	
C1	PVP combined with bilateral PPP	
D1	Unilateral novel bone cement bridging screw system combined with PVP	
E1	Bilateral novel bone cement bridging screw system combined with PVP	
A2	PVP alone	Axial compression test
B2	PVP combined with unilateral PPP	
C2	PVP combined with bilateral PPP	
D2	Unilateral novel bone cement bridging screw system combined with PVP	
E2	Bilateral novel bone cement bridging screw system combined with PVP	

**FIGURE 6:**
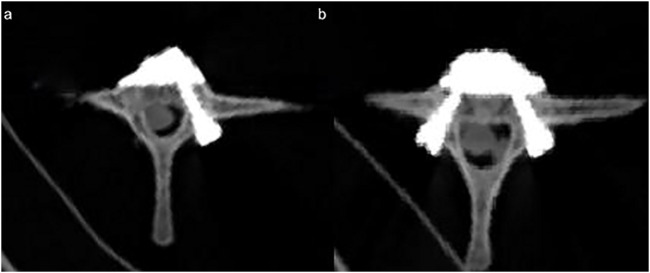
CT image after the placement of the novel bone cement screw: **(A)** unilateral novel bone cement bridging screw system combined with PVP; **(B)** bilateral novel bone cement bridging screw system combined with PVP.

At 24 h after preparation of specimen models, specimens were analyzed using CT (Figure 6) to determine the suitability of the screws and the presence of other injuries to the vertebral body. All specimens were subjected to three-dimensional biomechanical strength and maximum axial compression tests within 1 week after specimen acquisition.

### 2.5 Biomechanical experiments

Three-dimensional biomechanical strength test was conducted in six motion states including flexion, extension, lateral bending (right and left), axial rotation (right and left) on all specimen models in each group with six-axis spinal motion testing machine. Axial compression test was performed using microcomputer-controlled electronic universal testing machine. After the specimens were thawed at room temperature, the base of specimens was placed in the center of the fixture, and embedded with an appropriate amount of a mixture of polyol (Yishun Chemical Co., Ltd., Shenzhen, China) and isocyanate (Yishun Chemical Co., Ltd., Shenzhen, China). After waiting for about 10 min to allow the embedding medium to completely solidify, the embedded specimens were taken and stored at −25°C.

#### 2.5.1 Three-dimensional biomechanical strength test

**FIGURE 7:**
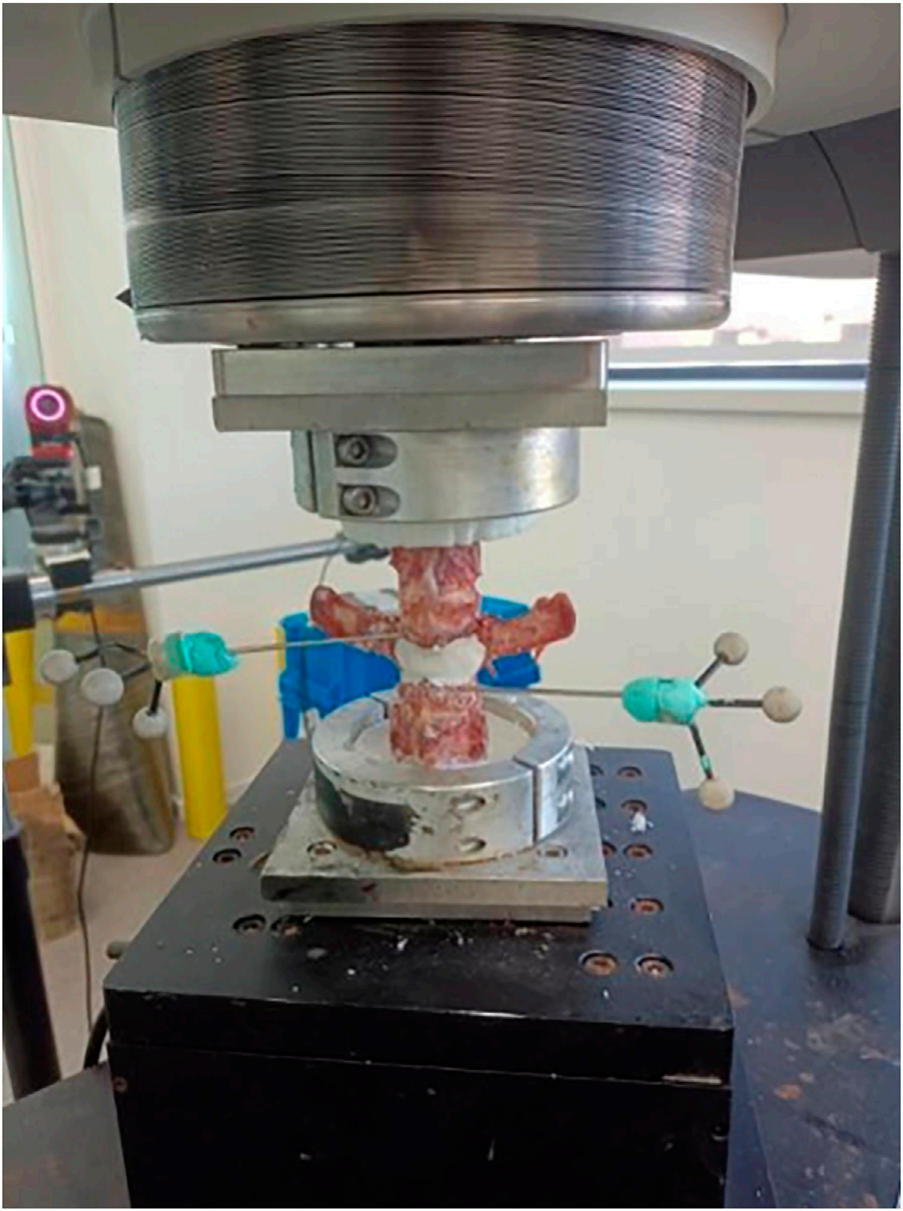
Three-dimensional biomechanical strength test.

Three-dimensional biomechanical strength test can effectively analyze the motion and strength of bone cement under simulated working conditions. The test was performed on all specimens with the same testing machine in a non-destructive manner. After the specimens were thawed at room temperature, one Kirschner wire (K-wire) was inserted at the superior and inferior edges of the cement-augmented area of the L1 vertebral body, and three light point balls of the OptiTrack^®^ motion capture system were subsequently fixed on the tip of the K-wire. The specimens of each group were placed on the biomechanical machine sequentially. In order to simulate the weight of the human upper body, a 500-N vertical compressive load was applied to the top of the specimens. Specimens were tested under six loading conditions (flexion, extension, left bending, right bending, and right/left axial rotation) with a moment of 10 Nm. The infrared camera of the OptiTrack^®^ motion capture system captured the light point balls fixed on the tip of the K-wire to record the three-dimensional motions (yaw, pitch, roll) of the superior and inferior K-wires. The overall deflection angles of the K-wires was then calculated by using the angle equation of the OptiTrack^®^ motion capture system, the difference between the deflection angles of the superior and inferior K-wires was used to represent the range of motion (ROM) in the cement-augmented area (Figure 7). During the test, specimens were kept moist through spraying of saline every 5 min. After the test was completed, CT examination was performed to evaluate the conditions of internal fixation and bone cement.

#### 2.5.2 Axial compression test

**FIGURE 8:**
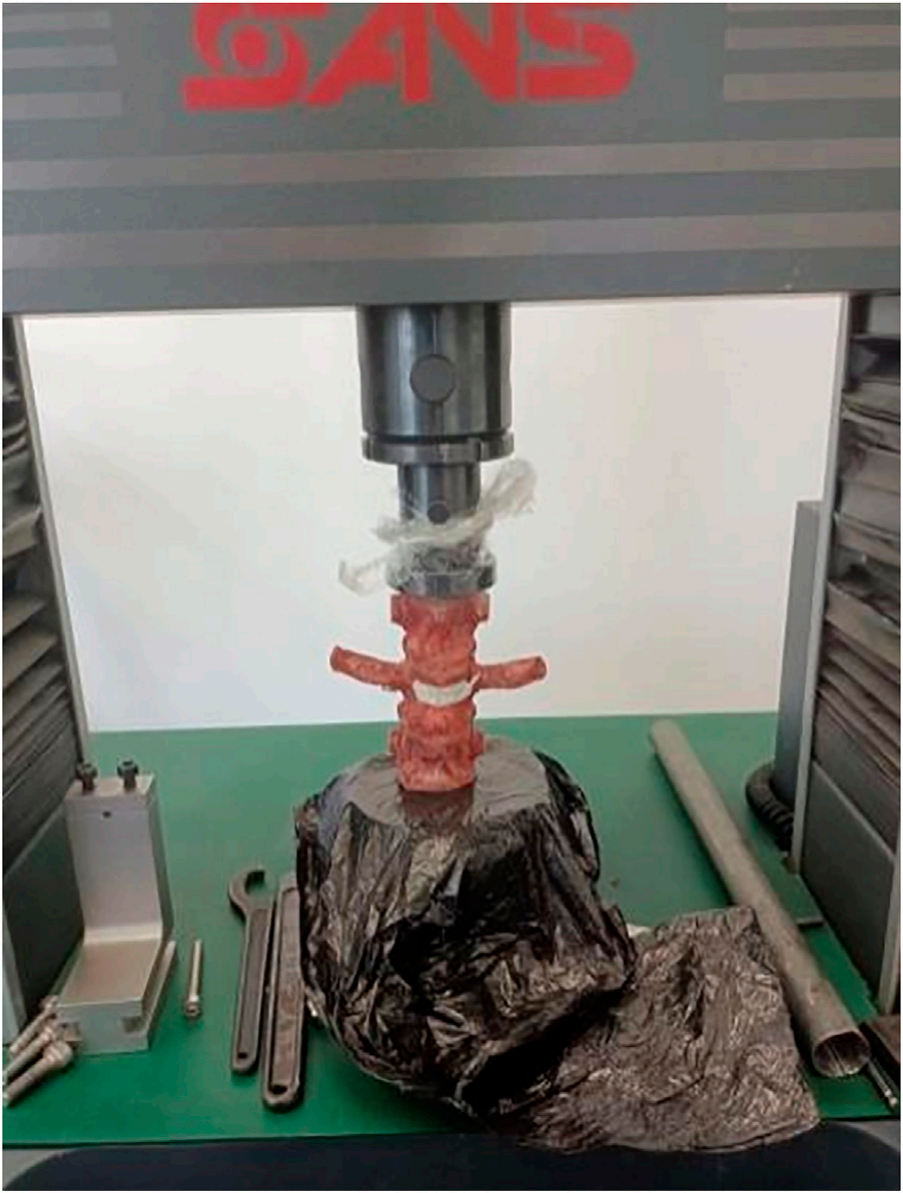
Axial compression test.

Axial compression test mainly tests the maximum load the bone cement can withstand under axial compression until the bone cement is loosened and displaced. The axial compression test was also performed on all specimens with the same microcomputer-controlled electronic universal testing machine. The specimens of each group were fixed on the testing machine sequentially, and the axial compression applied to the specimens was gradually increased until the bone cement appeared to be loosened and displaced. During the whole test, axial compression was applied to the specimens at a speed of 2 mm/min, with the maximum load-displacement set to 10 mm. The load curves of all specimens were observed until the peak load appeared (Figure 8).

### 2.6 Observation indicators

The motion of bone cement in all specimens of each group in six motion directions including flexion, extension, left bending, right bending, right/left axial rotation was analyzed in order to evaluate the three-dimensional strength of bone cement under different internal fixation conditions. The maximum axial pressure that the bone cement can withstand in axial compression until the occurrence of bone cement displacement was analyzed to evaluate the maximum axial pressure of the bone cement under different internal fixation conditions.

### 2.7 Statistical analysis

Statistical analysis was performed using the SPSS 26.0 statistical software package (IBM SPSS Inc., New York, United States). Data were expressed as mean ± standard deviation (SD). The Kruskal-Wallis test was used for multiple comparisons. The Wilcoxon rank sum test was used for comparisons between two groups. A *p* < 0.05 was regarded as statistically significant. All statistical tests were two sided and the significance level (α) was set at 0.05.

## 3 Results

### 3.1 Results from three-dimensional biomechanical strength test

**FIGURE 9:**
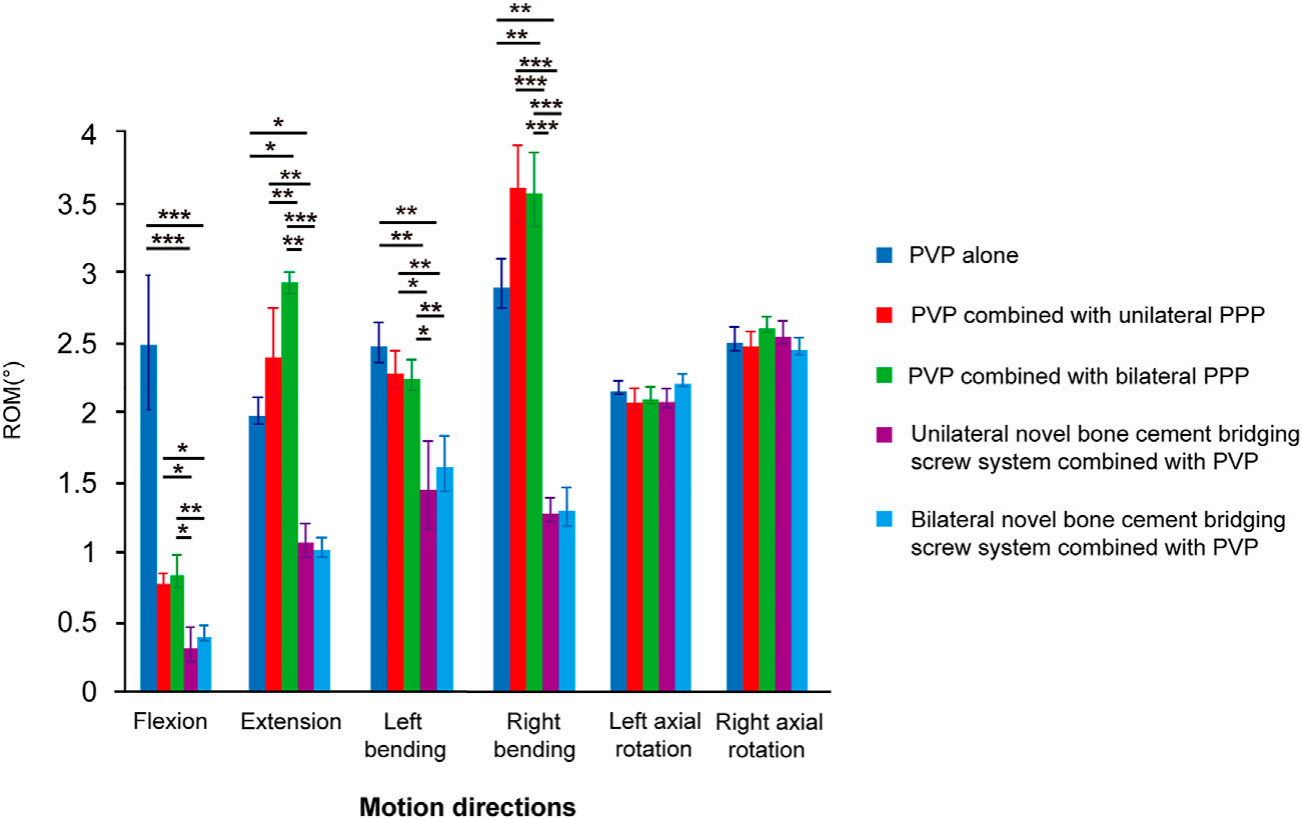
ROM of five group in six motion directions. *p < 0.05, **p < 0.01, ***p < 0.001.

ROM of the bone cement-augmented models in 6 six motion directions including flexion, extension, left bending, right bending, right/left axial rotation was compared between the five groups. The results showed that there were statistically significant differences in the motion of bone cement in four motion directions including flexion, extension, left bending, right bending between the five groups (*p* < 0.05), whereas the motion of bone cement in left and right axial rotation did not differ significantly between the five groups (*p* > 0.05). Furthermore, pairwise comparison showed that the motion of bone cement in four motion states including flexion, extension, left bending, right bending was significantly smaller, and the biomechanical strength of bone cement was better in both the unilateral and bilateral novel bone cement bridging screw system combined with PVP groups than in other three groups (*p* < 0.05), whereas no significant difference was found between the unilateral and bilateral novel bone cement bridging screw system combined with PVP groups (*p* > 0.05). The results suggest that unilateral and bilateral novel bone cement screw placement have little influence on the biomechanical strength of bone cement, and can both achieve better results. However, there was no significant difference in the motion of bone cement between groups in two motion states including left and right axial rotation (*p* > 0.05) (Table 2; Figure 9).

**TABLE 2:** Comparison of the three-dimensional biomechanical strength of five group (°).

Group	Flexion	Extension	Left bending	Right bending	Left axial rotation	Right axial rotation
A1	2.48 ± 0.48^a^ ^,^ ^b^	1.98 ± 0.10^a^ ^,^ ^b^	2.47 ± 0.14^a^ ^,^ ^b^	2.92 ± 0.18^a^ ^,^ ^b^	2.18 ± 0.05	2.52 ± 0.09
B1	0.80 ± 0.04^a^ ^,^ ^b^	2.39 ± 0.32^a^ ^,^ ^b^	2.28 ± 0.13^a^ ^,^ ^b^	3.63 ± 0.28^a^ ^,^ ^b^	2.10 ± 0.08	2.50 ± 0.08
C1	0.86 ± 0.11^a^ ^,^ ^b^	2.92 ± 0.08^a^ ^,^ ^b^	2.24 ± 0.11^a^ ^,^ ^b^	3.59 ± 0.27^a^ ^,^ ^b^	2.12 ± 0.06	2.62 ± 0.06
D1	0.34 ± 0.12	1.08 ± 0.12	1.46 ± 0.31	1.30 ± 0.09	2.10 ± 0.07	2.57 ± 0.08
E1	0.42 ± 0.06	1.03 ± 0.07	1.61 ± 0.19	1.32 ± 0.14	2.23 ± 0.04	2.47 ± 0.06

Compared with D1.

^a^ *P* < 0.05(Wilcoxon test); Compared with E1.

^b^ *P* < 0.05(Wilcoxon test).

### 3.2 Results from axial compression test

**FIGURE 10:**
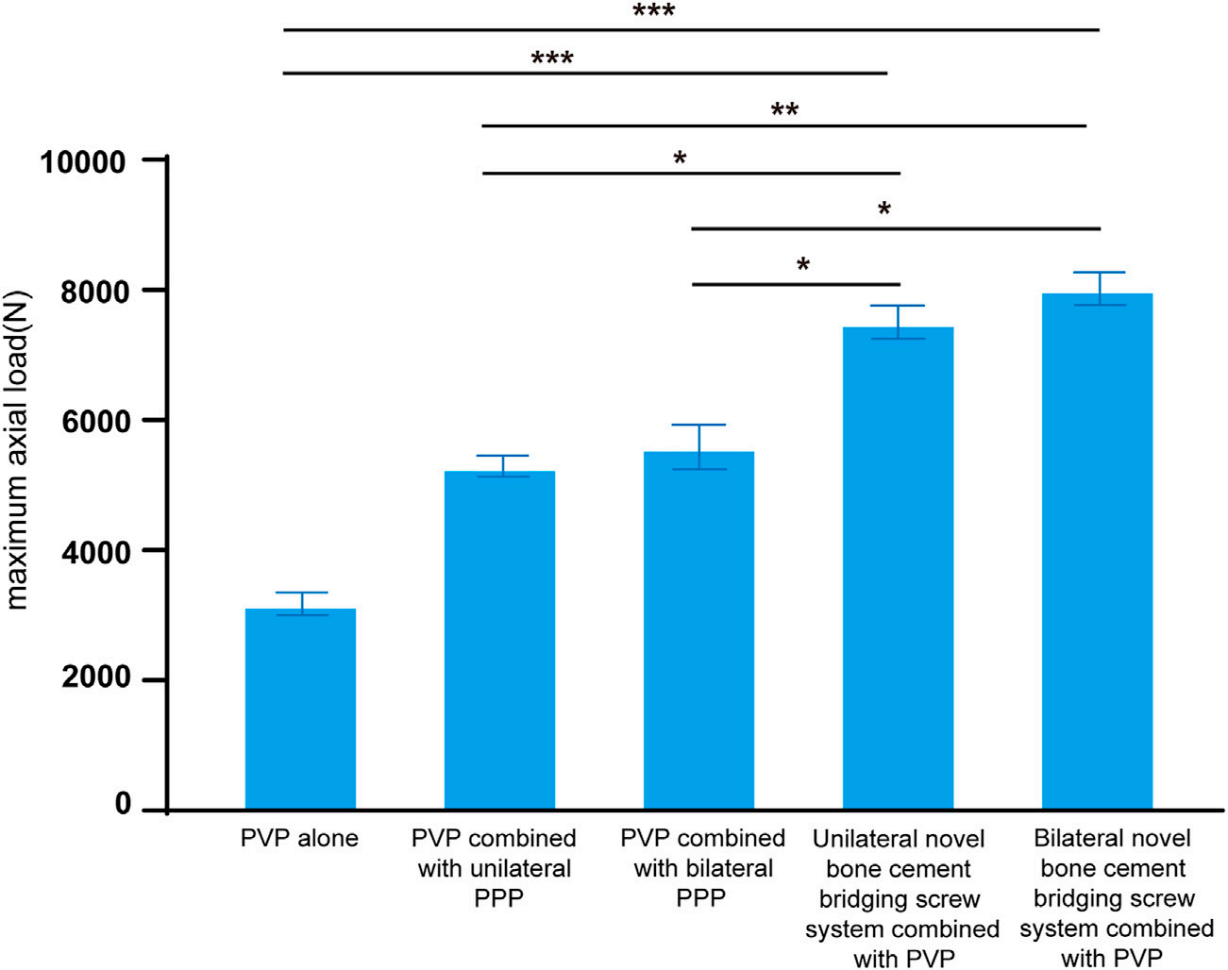
Maximum axial load of five group. *p < 0.05, **p < 0.01, ***p < 0.001.

Comparison of the maximum axial load of the bone cement-augmented models in axial compression between the five groups showed that the maximum axial load of bone cement was greater, with better strength in both the unilateral and bilateral novel bone cement bridging screw system combined with PVP groups compared with other three groups (*p* < 0.05), whereas there was no significant difference in the maximum axial load of bone cement between the unilateral and bilateral novel bone cement bridging screw system combined with PVP groups (*p* > 0.05). The results indicate that both unilateral or bilateral screw placement can withstand similar load under vertical forces, show better load carrying capacity, and exhibit a better ability to avoid cement loosening and displacement (Table 3; Figure 10).

**TABLE 3:** Comparison of the maximum axial pressure of five group (N).

Group	Maximum axial load
A2	3,176.72 ± 174.88^a^ ^,^ ^b^
B2	5,290.25 ± 161.91^a^ ^,^ ^b^
C2	5,587.96 ± 343.99^a^ ^,^ ^b^
D2	7,508.28 ± 255.38
E2	8,018.98 ± 249.66

Compared with D2.

^a^ *P* < 0.05(Wilcoxon test); Compared with E2.

^b^ *P* < 0.05(Wilcoxon test).

## 4 Discussion

### 4.1 Problems in the treatment of KD

The use of bone cement to effectively fill the IVC to reconstruct the strength of the vertebral body is the most safe and minimally invasive treatment for KD. However, vertebroplasty is associated with a cement loosening rate of up to 45%, which can lead to the reoccurrence of severe pain, and even the development of bone cement displacement in severe cases (Nakamae et al., 2018). Worldwide, the causes of bone cement displacement after vertebroplasty for KD are currently unknown, there are no practical solutions to this problem, and methods to avoid loosening and displacement of bone cement after vertebroplasty have been rarely reported, technical gap regarding this aspect exists.

PMMA-based bone cement has been widely used in spinal surgery and remains the most convenient material for filling and reconstruction of the vertebral body (Li et al., 2017). However, due to the high strength of PMMA, the weak bonding between the cancellous bone and PMMA bone cement, and some serious consequences caused by bone cement loosening and displacement, the use of PMMA bone cement is controversial. A study conducted by Lee et al. (2011) showed that when IVC exists, PMMA cannot provide proper support for reconstruction of the diseased vertebral body, because PMMA cannot intermingle with vertebral trabeculae, which may cause the possible displacement of PMMA into the spinal canal, thus leading to neurological deficits. Currently, some scholars recommend percutaneous bone cement-augmented pedicle screw fixation as an effective treatment for KD with multiple comorbidities and/or severe osteoporosis, which can also be used to treat KD with myelopathy, spinal cord compression, spinal instability, and without neurological deficits (Chen et al., 1976; Mohan et al., 2012; Zhang et al., 2013; Kühn and Höntzsch, 2015; Park et al., 2015; Huang et al., 2018; Lim et al., 2018), but this treatment method has the disadvantages of loss of motion segments, limited ROM of the thoracic and lumbar spine, accelerated degeneration of adjacent segments, and high cost. Wang et al. (2020) also proposed the use of robot-assisted PVP combined with PPP for single-segment KD, but there is a risk of bone cement leakage into spinal canal or intervertebral foramen. In recent years, there have been some new devices for assisted-vertebroplasty, such as Kiva (Benvenue Medical, United States), SpineJack (Vexim, France), V-STRUT (Hyprevention, France) or SAIF (DePuySynthes-Johnson and Johnson, United States) (Tutton et al., 1976; Aebi et al., 2018; La Barbera et al., 2019a; Hartman et al., 2019). However, the purpose of these new devices is to provide better reduction of fractured vertebral bodies, and prevent loss of reduction vertebral height and progression of post-PVP fractures, rather than for IVC treatment to avoid loosening and displacement of bone cement.

At present, there is still a large technical gap for solving the problem of bone cement loosening and displacement. The primary reason is that the bone cement in the IVC cannot be integrated with the surrounding trabecular bone tissue. Based on this aspect, our team innovatively designed a novel bone cement bridging screw system to treat KD in combination with PVP, in order to lock the bone cement and the surrounding bone tissue together, thereby avoiding the occurrence of bone cement loosening and displacement during and after surgery. The novel bone cement bridging screw system adopts a unique inner and outer dual-thread design without end cap, the bone cement injection device is tightly connected with the inner thread of the screw, and there is a unique hollow channel inside the screw for bone cement injection and multiple bone cement outlets on the tip of the screw, so the novel bone cement screw allows integration between bone cement and the screw during bone cement injection. Additionally, the screw is placed in the vertebral body and pedicle, which acts as a “bridge” to interlock bone cement and the surrounding bone tissue, and even the pedicle (a strongest point of attachment of the spine), thereby creating a tight interlock between bone cement and the vertebral body. Furthermore, the screw is less likely to be loosened and displaced. In the present study, we analyzed the biomechanical strength of this novel bone cement bridging screw system for KD models in T12-L2 sheep spine specimens, we hope this work will provide mechanical foundation for future studies in humans and clinical promotion.

### 4.2 Biomechanical strength of the novel bone cement bridging screw system in flexion and extension

The three-dimensional biomechanical strength test showed that the biomechanical strength of bone cement in two motion directions including flexion and extension in the unilateral and bilateral novel bone cement bridging screw system combined with PVP was better compared with other three groups, and statistically significant differences were noted in the strength of bone cement among the five groups under the same experimental conditions (*p* < 0.05). Multiple comparisons between groups revealed that both unilateral and bilateral novel bone cement bridging screw system combined with PVP were better than other three internal fixation modalities in improving the strength of bone cement during flexion and extension motions (*p* < 0.05). The results suggest that either unilateral or bilateral novel bone cement screw placement combined with PVP could markedly enhance the strength of bone cement during flexion and extension motion of the spine after bone cement augmentation for KD, and achieve similar results. We believe that the main reason is that the ingenious design of the novel bone cement bridging screw system allows for rapid injection of bone cement through the hollow channel inside the screw, thus enabling better integration between the bone cement and the screw. And the screw is embedded into the vertebral body, which allows the screw to be tightly interlocked with the bone tissue, makes the originally weak bond between PMMAbone cement and cancellous bone more stable through the bridge action of the novel bone cement bridging screw system. Additionally, during the flexion and extension of the spine, the screw and bone cement are interpreted moving synchronously within the vertebral body, which do not affect the vertebral body movement, so that the screw, bone cement, vertebral body are integrated and become a whole, thus achieving better strength.

### 4.3 Biomechanical strength of the novel bone cement bridging screw system in bending

Under the same conditions, bone cement strength in left and right bending was significantly better in both the unilateral or bilateral novel bone cement bridging screw system combined with PVP groups than in other 3 groups (*p* < 0.05). The results indicate that the novel bone cement bridging screw system can obviously enhance the strength of bone cement in left and right bending motions after bone cement augmentation for KD, by which the strength of bone cement was increased by 28.15%–64.19%. However, no statistically significant difference was found between the unilateral or bilateral novel bone cement bridging screw system combined with PVP groups (*p* > 0.05), indicating that both unilateral and bilateral novel bone cement screw placement can achieve the same effect for stabilization of bone cement during lateral bending motion. The authors believe that this may be due to that the novel bone cement screw has multiple lateral bone cement outlets on its tip, enabling close and firm integration of the screw with bone cement 360° around the screw. During left and right bending, the screw can still firmly interlock with bone cement, and better withstand lateral bending pressure.

### 4.4 Biomechanical strength of the novel bone cement screw in axial rotation

No significant difference was noted in the bone cement strength in left and right axial rotation between the five groups (*p* > 0.05), indicating that the novel bone cement bridging screw system does not obviously improve the strength of bone cement in axial rotation, this is a disadvantage of the novel bone cement screw. The authors speculate that this may be due to that the position of the bone cement screw is parallel to the axial rotational plane, the screw and the cement can rotate simultaneously when the vertebral body rotates, and the screw does not influence the rotational movement of bone cement, so the bone cement strength in left and right axial rotation did not differ significantly between the use of novel bone cement screw placement and other internal fixation modalities. Additionally, these five bone cement augmentation modalities do not fix vertebral motion segment, and do not influence the rotation of the diseased vertebrae, this is therefore an important theoretical reason why bone cement strength in left and right rotation do not vary considerably among these five fixation modalities.

### 4.5 Biomechanical strength of the novel bone cement screw in axial compression

The axial compression test showed that the specimens in the unilateral and bilateral novel bone cement bridging screw system combined with PVP groups were able to withstand greater axial load, and were less likely to develop bone cement loosening and displacement than specimens in other three groups (*p* < 0.05). Unilateral or bilateral novel bone cement screw placement combined with PVP exhibited better load carrying capacity than other fixation modalities. Compared to the use of PVP alone, the load carrying capacity of unilateral and bilateral novel bone cement screws combined with PVP was increased by 136.35% and 152.43%, respectively. This result demonstrates that the novel bone cement screw have a unique structure that enables better interlock between the screw and bone cement, enhances its ability to maintain vertebral body height, and allows the bone cement-augmented vertebral body to sustain greater load without the occurrence of bone cement loosening and displacement. In clinical practice, use of this novel bone cement bridging screw system could restore weight-bearing function of the spine in KD patients. The results of the present study demonstrate that the novel bone cement bridging screw system can better stabilize the bone cement, thus preventing bone cement loosening and displacement.

### 4.6 Study limitations

The study has certain limitations. First, the specimens used in the study was obtained from the sheep spine, which differ somewhat from humans, and may not exactly match the physiological and mechanical properties structures of human spine. Second, the number of specimens used in the study was small, more data are needed to support and verify the results. In addition, *ex vivo* specimens were used in the study, results obtained from mechanical tests only reflected the immediate strength, which cannot reflect the long-term mechanical strength. Meanwhile, all the muscles were removed from all the specimens, and only the posterior ligaments, joint capsules, intervertebral discs and all bony structures were preserved, which did not fully simulate the real situation *in vivo*. Finally, the specimens used in the study were all normal vertebral bodies without injury and osseous abnormalities, but in actual conditions, KD are mostly associated with osteoporosis and worse vertebral body strength, so further studies using human cadaveric specimens are needed to confirm whether the same immediate strength can be achieved with the novel bone cement bridging screw system.

## 5 Conclusion

Through three-dimensional biomechanical strength test and axial compression test on sheep vertebral specimens, our findings demonstrate that the novel bone cement bridging screw system could obviously improve the strength of bone cement in four motion directions including flexion, extension, left bending, right bending, enable the bone cement to sustain greater axial pressure in the axial direction, thereby avoiding the catastrophic complications including bone cement loosening and displacement to the greatest extent in the treatment of KD.

## Data Availability

The raw data supporting the conclusion of this article will be made available by the authors, without undue reservation.
